# Supplementary material for: Diagnosis and Treatment of Children with a Radiological Fat Pad Sign without Visible Elbow Fracture Vary Widely: An International Online Survey and Development of an Objective Definition
Source: Children (Basel). 2022 Jun 25;9(7):950. doi: 10.3390/children9070950 (PMC9319871; doi:10.3390/children9070950)
Supplement: Supplementary file 1 [file children-09-00950-s001.zip › children-1772478-supplementary.pdf]

Supplementary Materials:

The online survey

Demographics

1 In which country are you currently working?

Drop-down menu

2 How many paediatric elbow injuries do you treat annually?

<10 | 10-20 | 20-50 | >50

3 What is your expertise?

Orthopaedic resident
Resident in trauma surgery
Orthopaedic upper limb surgeon
Orthopaedic trauma surgeon
Paediatric orthopaedic surgeon
General orthopaedic surgeon
Trauma surgeon
Other

4 If you entered 'other' in the previous question, please explain your expertise here:

Open

5 How many years have you been in practice as an (orthopaedic or trauma) surgeon?

0 | 1-5 | 6-10 | >10

Definition

6 How would you define an anterior fat pad sign on the lateral elbow radiograph?

Open

7. How would you define a posterior fat pad sign on the lateral elbow radiograph?

Open

Probability diagnostics

8. What is the probability of an occult fracture in case of a positive FPS?

Slide bar from 0-100%

9. What is the most probable diagnosis?

Lateral condyle fracture | Medial epicondyle fracture
Olecranon fracture | Radial head fracture
Radial neck fracture | Supracondylar fracture
Other; pass through dislocation (and immediate reduction)

10. If you entered 'other' in the previous question, name the location of the most probable fracture or lesion:

Open

Open

Further diagnosis

11. What is your usual further diagnostic workup?

No further imaging
Additional radiographs in 2 directions (2 additional oblique views)

Repeat radiographs on indication
Repeat radiographs after 1 week
CT
MRI
Other

12. If you entered 'other' in the previous question, please explain your further diagnostic workup here:
Open

Standard treatment

13. What is your standard treatment in case of a positive fat pad sign without visible fracture?
Functional treatment (i.e. no immobilization)
Pressure bandage
Plaster/casting
Sling
I have no standard treatment
Other

14. If you entered 'other' in the previous question, please explain your standard treatment here:
Open

Follow-up

15. How is the follow-up organized?
No follow-up
1 Week
2 Weeks
3 Weeks
Depending on additional imaging

16. If you entered 'other' in the previous question, please explain your follow-up treatment here:
Open

Radiographs

17-56. There will be posted 20 radiographs here, to be assessed for the presence or absence of anterior (yes / no) and or posterior fat pad sign (yes / no).
